# Pneumoperitoneum in Rectal Polyembolokoilamania

**DOI:** 10.7759/cureus.19697

**Published:** 2021-11-18

**Authors:** Joel Thomas, Sagar Maheshwari, Ahmed Sallam

**Affiliations:** 1 Radiology, Barking, Havering and Redbridge University Hospitals NHS Trust, London, GBR; 2 Internal Medicine, Leicester Royal Infirmary, Leicester, GBR

**Keywords:** psychiatry, pestle, foreign body insertion, rectal polyembolokoilamania, pneumoperitoneum

## Abstract

Foreign body insertion in the rectum is not a very common presentation in the emergency department but this is common among individuals with a history of self-harm, personality disorders, and other psychosomatic illnesses. It is often diagnosed on abdominal x-rays; however, a CT scan of the abdomen and pelvis may be warranted when perforation is suspected. To diagnose an anorectal foreign body, clinicians must maintain a high level of suspicion. Because of embarrassment or maybe psychological concerns, the patient may not be ready to share all the information. Healthcare providers must hence show empathy and compassion while being calm and non-judgmental. Here, we present a case of an 80-year-old male who underwent a laparotomy for removal of a large foreign body that was inserted in the rectum and caused a gastrointestinal perforation.

## Introduction

Anorectal damage caused by foreign body (FB) insertion has been documented since the 1500s. Since then, there have been many reports in the literature of various objects being pushed into the anus. A wide variety of objects self-inserted into the urinary tract have been reported and that includes electrical wires, batteries, glass, pencils, chopsticks, and telephone cables as opposed to anorectal objects documented such as lightbulbs, sex toys, toothbrushes, drugs, cell phones, fruits, vegetables, and in one incidence a frozen pig's tail [1-8]. Fear of social embracement may force a patient to procrastinate medical assessment and therapy, thereby leading to complicating management protocols. As a result, we emphasize the necessity of a high level of suspicion, as well as a thorough and extensive evaluation, followed by a patient-specific strategy through this case report.

## Case presentation

An 80-year-old male patient presented to the accident and emergency department (A&E) with sudden acute abdominal pain, distension, and confusion. No surgical scars were noted on his abdomen and his abdomen was clinically distended. He had no past medical history correlating to his presentation. The patient was hemodynamically stable and was admitted to the hospital for further investigations. The role of psychological factors was not considered at this moment, as he shared no past medical history of the same. At this stage, we clinically suspected intestinal obstruction, perforation, or pancreatitis. Upon doing a CT abdo-pelvis later, a pestle-like object was found in the rectum causing perforation. He later admitted that he fantasizes putting objects into his body orifices, and had been diagnosed with polyembolokoilamania before. He initially developed the habit of inserting small objects and then proceeded to try out bigger objects with time.

**Figure 1:**
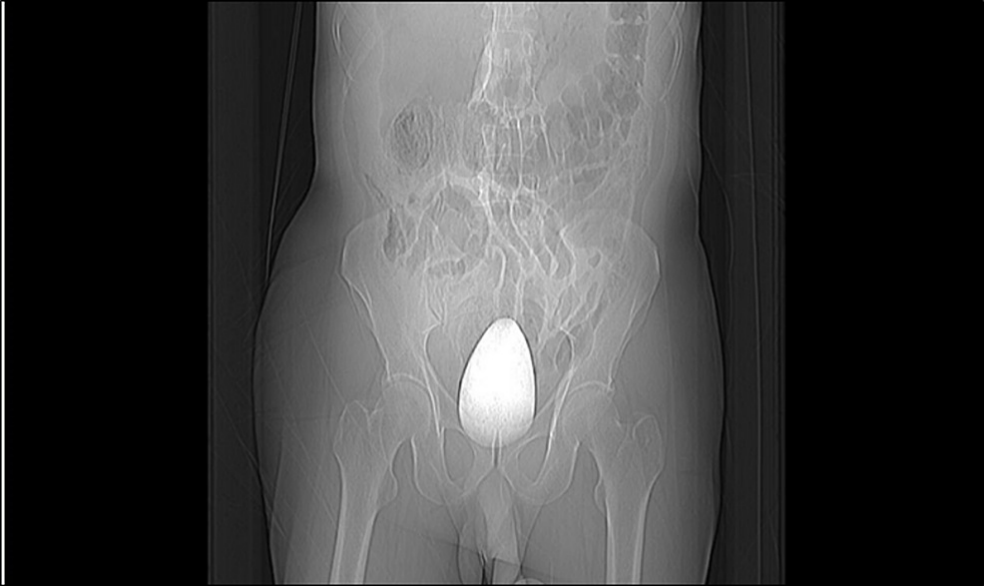
Abdominal x-ray showing the presence of a large foreign body in rectum.

**Figure 2:**
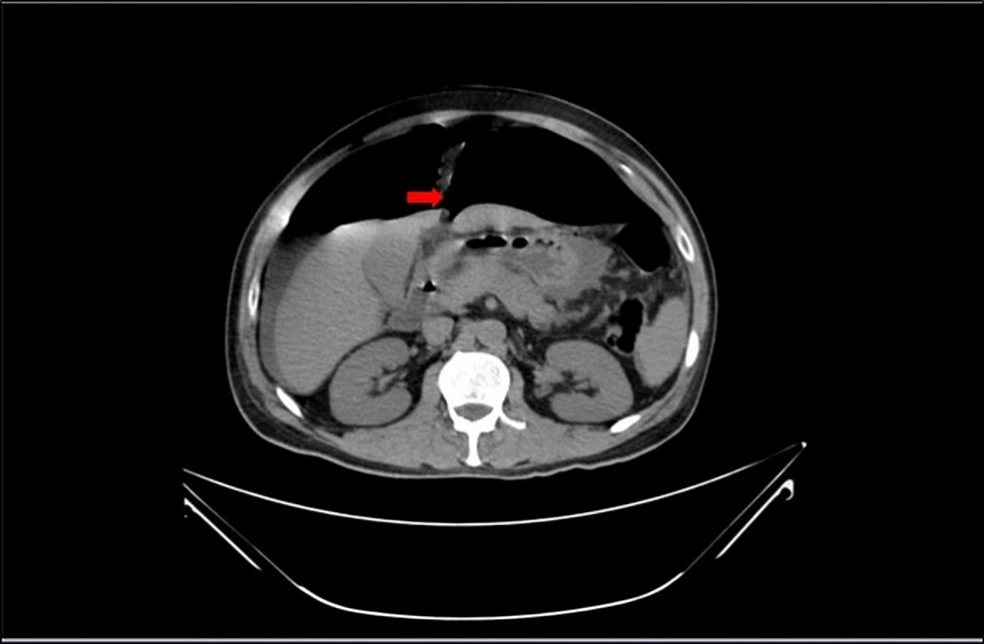
CT abdomen - transverse view showing falciform ligament sign.

**Figure 3:**
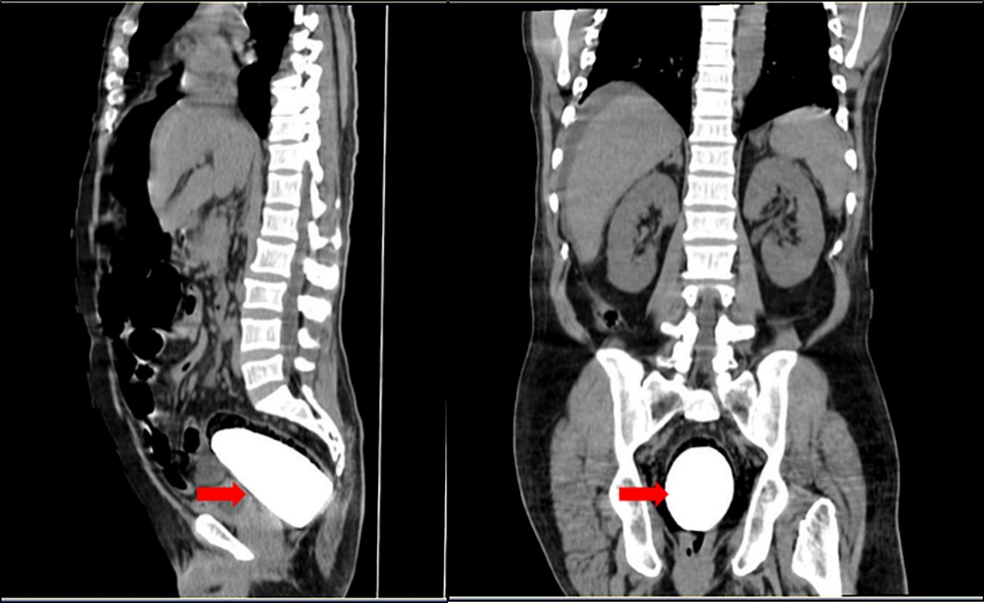
CT abdomen - sagittal and coronal views demonstrating what looks like a "pestle" in the rectum.

An initial abdominal x-ray confirmed the presence of a large foreign body in rectum (Figure 1). An urgent abdominal ultrasonography performed showed gaseous abdomen, suspecting free air, but it was inconclusive due to reverberation artifacts. CT abdomen done thereafter revealed an oblong cylindrical shaped object in the rectum causing proximal colon dilatation and rectal perforation, along with free fluid collection. Various signs of pneumoperitoneum, such as the falciform ligament sign, were noted on the CT (Figures 2, 3).

The patient underwent a surgical laparotomy to remove the object, where it was then confirmed that it was a ceramic pestle. The patient recovered after surgery from acute abdominal perforation and was transferred for care under the psychiatry team.

## Discussion

Polyembolokoilamania represents a broad group of disorders characterized by the self-insertion of objects into body orifices caused by a number of psychosocial and psychological conditions. It is common in people who have a history of self-harm, personality disorders, schizophrenia, or alcoholism, as well as in prison inmates [9]. Adults who insert foreign objects, and often suffer from mental illness, have residual curiosities that manifest as experimentation, attempts to rekindle past memories or relationships, or do so to increase sexual arousal which is the most common motive [10,11]. The majority of patients present after attempting to remove the object at home but failing or when it has caused perforation. Patients frequently give vague history as it was with this case. The situation is further complicated by the delayed presentation, which is sometimes due to embarrassment, or the varied presentations of the numerous items of various sizes and shapes that find their way into one's rectum [9,12]. In patients presenting with acute abdominal pain with or without psychiatric history, foreign bodies should always be part of the differential diagnosis. Rectal foreign bodies can be detected by either x-rays or CTs. If perforation is ever suspected, in cases like peritonitis or shock, CT should be done, preferably with rectal contrast or rectal enema with water-soluble contrast to detect the level of the perforation. No attempts at bedside extraction should be made if peritonitis is present or if the patient is unstable, instead, the patient should be moved to the operating room for an emergent laparotomy. Prior to surgery, intravenous fluids and antibiotics should be administered. If a perforation is suspected, the presence of pneumoperitoneum on a clear abdominal film can quickly validate the diagnosis and speed up the decision to operate. In the vast majority of patients, however, these signs may not be present and a less invasive method can be attempted [13].

## Conclusions

One should always keep a high index of suspicion of the psychological factors that may have an indirect role in acute abdomen presentations, through the insertion of foreign bodies, even when there is no clear history of any psychiatric illness. In most cases, plain abdominal x-ray may be sufficient, but in cases where perforation is suspected, computed tomography is preferred. Various treatment options such as manual evacuation of foreign body, sigmoidoscopic removal, or laparotomy may be considered on a case-to-case basis. A detailed psychiatric evaluation is also warranted after the surgical management.
